# Supplementary material for: Evidence of an allostatic response by intestinal tissues following induction of joint inflammation
Source: PLoS One. 2026 Jan 23;21(1):e0338053. doi: 10.1371/journal.pone.0338053 (PMC12829947; doi:10.1371/journal.pone.0338053)
Supplement: S1 Fig — (A) Schematic of in vivo CFA Model (see Methods for Details). Rats received one intra-articular injection (IAI) of 50 µL of CFA (10 mg/ml) or sterile saline into both knee joints (B). Number of rats used for each study group (Naïve, Saline, CFA). Experiments were performed on 2 separate cohorts but procured from the same supplier. S3 = Proximal Ileum (PI), S4 = Distal Ileum (DI) C = Colon (Co). (C) Segmentation of small intestine and colon for RNASeq analyses and histological evaluations (see Methods for details). S = small intestine, S3 = proximal Ileum, S4 = distal Ileum, C = distal colon. (PPTX) [file pone.0338053.s001.pptx]

## Slide 1
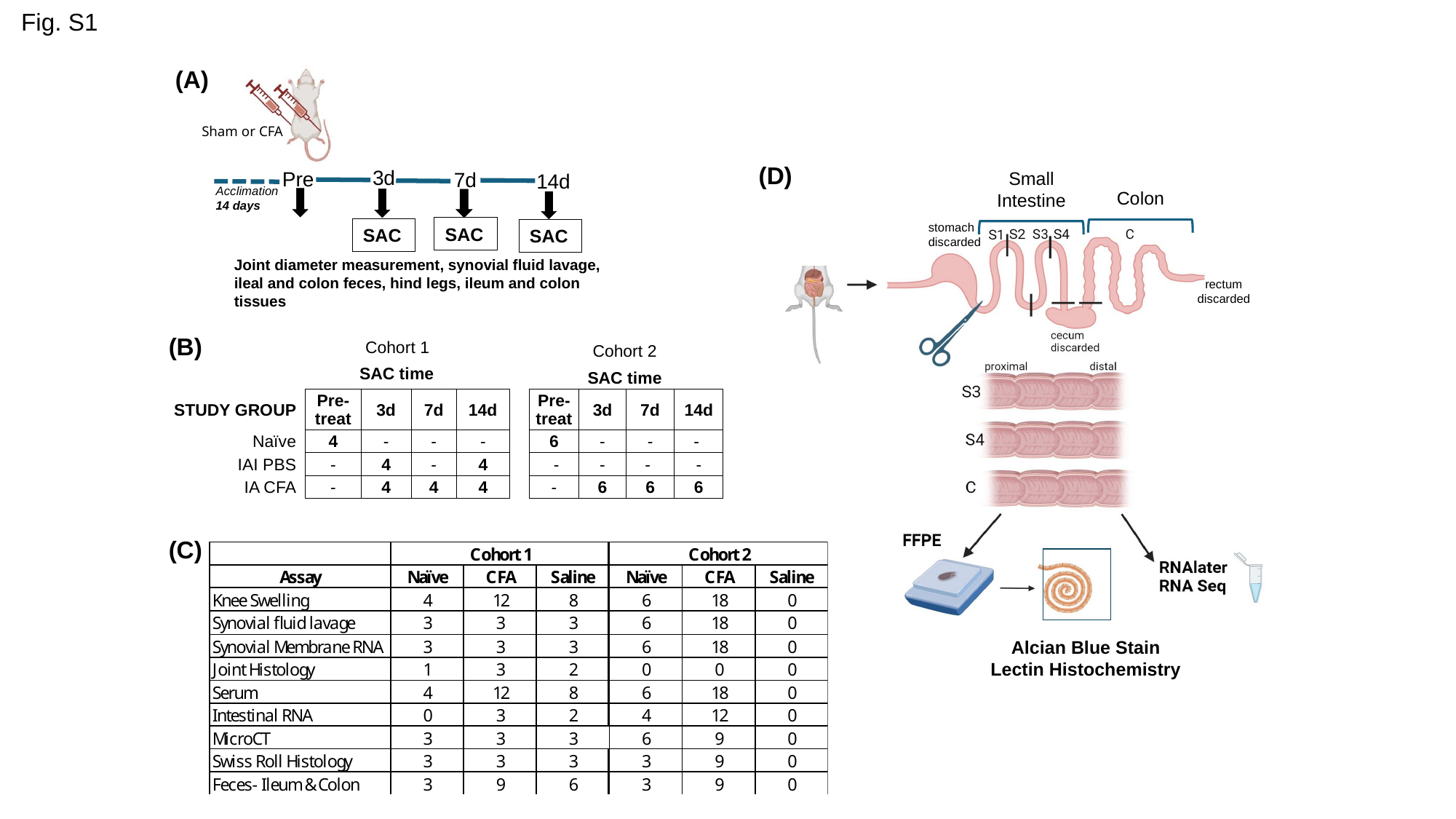

Fig. S1
(A)
Sham or CFA
3d
Pre
7d
14d
Acclimation
14 days
SAC
Joint diameter measurement, synovial fluid lavage, ileal and colon feces, hind legs, ileum and colon tissues
(D)
Small Intestine
Colon
stomach
discarded
SAC
SAC
rectum
discarded
(B)
Cohort 1
Cohort 2
SAC time
SAC time
| STUDY GROUP | | Pre- treat | 3d | 7d | 14d | | Pre- treat | 3d | 7d | 14d |
| --- | --- | --- | --- | --- | --- | --- | --- | --- | --- | --- |
| Naïve | | 4 | - | - | - | | 6 | - | - | - |
| IAI PBS | | - | 4 | - | 4 | | - | - | - | - |
| IA CFA | | - | 4 | 4 | 4 | | - | 6 | 6 | 6 |
(C)
Alcian Blue Stain
Lectin Histochemistry
