# Supplementary material for: Evidence of an allostatic response by intestinal tissues following induction of joint inflammation
Source: PLoS One. 2026 Jan 23;21(1):e0338053. doi: 10.1371/journal.pone.0338053 (PMC12829947; doi:10.1371/journal.pone.0338053)
Supplement: S2 Fig — (A) A Western Blot of mouse serum treated with/without chondroitinase ABC lyase (Ch’ase) or NaOH to release bikunin or bikunin•CS, respectively from IαI, PαI and bikunin•HS species was used to characterize a rabbit anti-mouse bikunin antibody (denoted ap_A_mBikunin) as described in the Methods. Note that the mouse sequence of the immunizing peptide, AVLPQESEGS, is highly similar to the corresponding rat sequence, AVLPQENEGS, with only one amino acid difference. (B) Western blot of rat sera, collected from 4 individual rats prior to IAI-CFA, and mouse serum (as a standard) were prepared and treated without (lanes 1–4) or with (lanes 5–8) chondroitinase ABC lyase (Ch ABC) prior to electrophoresis as described in the Methods. (C) Western blot of rat sera of knee joint synovial fluid lavages collected from 3 individual rats at d3 post IAI-CFA and ‘standard’ mouse serum were prepared and treated or not with chondroitinase ABC (Ch ABC) prior to electrophoresis as described in the Methods. Positions of bikunin-contianing species in B and C (i.e., IαI, PαI, bikunin-CS and bikunin) are indicated to the right of the blots. All membranes (in A, B, C) were incubated with ap_A_mBikunin as described in the Methods. (D) To assess effusion of serum proteins into the joint space following IAI-CFA, portions of rat synovial fluid lavages collected prior to IAI-CFA (d0) and at d3, d7 and d14 post IAI-CFA, and rat serum pre-IAI-CFA, were electrophoresed with or without prior treatment with chondroitinase ABC (Ch ABC), and gels were Coomassie stained. Expected migration positions of lactoferrin, albumin and IgG are shown to the right. (PPTX) [file pone.0338053.s002.pptx]

## Slide 1
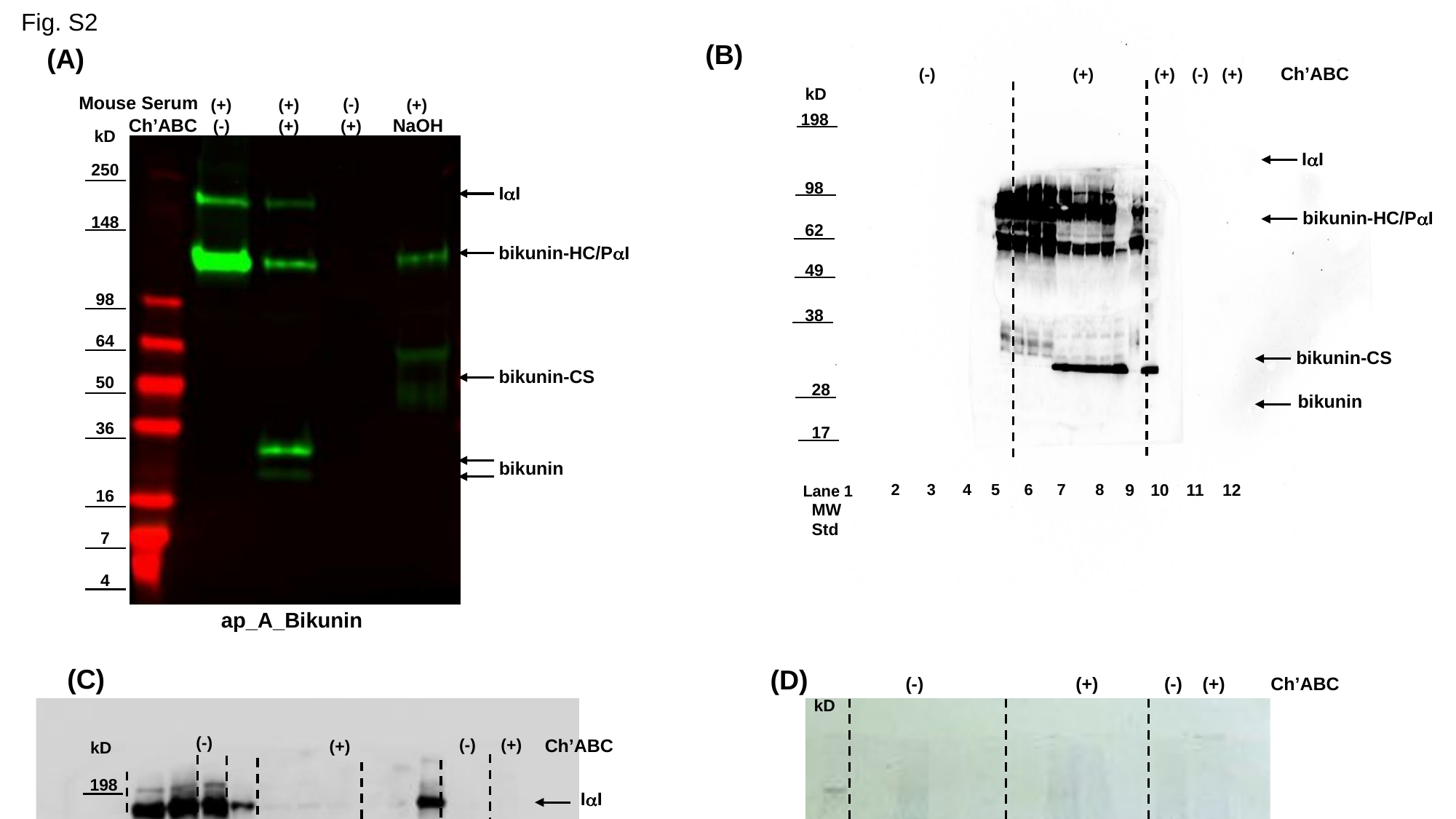

bikunin-CS
bikunin
Fig. S2
(B)
(A)
Ch’ABC
(-)
(+)
(+)
(-)
(+)
kD
Mouse Serum
(-)
(+)
(+)
(+)
Ch’ABC
NaOH
(-)
(+)
(+)
250
IaI
148
bikunin-HC/PaI
98
64
bikunin-CS
50
36
bikunin
16
7
4
198
kD
IaI
98
bikunin-HC/PaI
62
49
38
28
17
2
3
4
5
6
7
8
9
10
11
12
Lane 1
MW
Std
ap_A_Bikunin
(C)
(D)
 (-) (+) (-) (+) Ch’ABC
kD
(-)
(-)
(+)
Ch’ABC
(+)
kD
198
IaI
98
bikunin-HC/PaI
98
62
Lactoferrin
49
62
Albumin
38
IgG
bikunin-CS
28
bikunin
MW
Std
MW
Std
Lane 1
12
2
3
4
5
6
7
8
9
10
11
Rat Serum
d0
d0
d3
d7
d14
d3
d7
d14
