# Supplementary material for: Evidence of an allostatic response by intestinal tissues following induction of joint inflammation
Source: PLoS One. 2026 Jan 23;21(1):e0338053. doi: 10.1371/journal.pone.0338053 (PMC12829947; doi:10.1371/journal.pone.0338053)
Supplement: S3 Fig — (PPTX) [file pone.0338053.s003.pptx]

## Slide 1
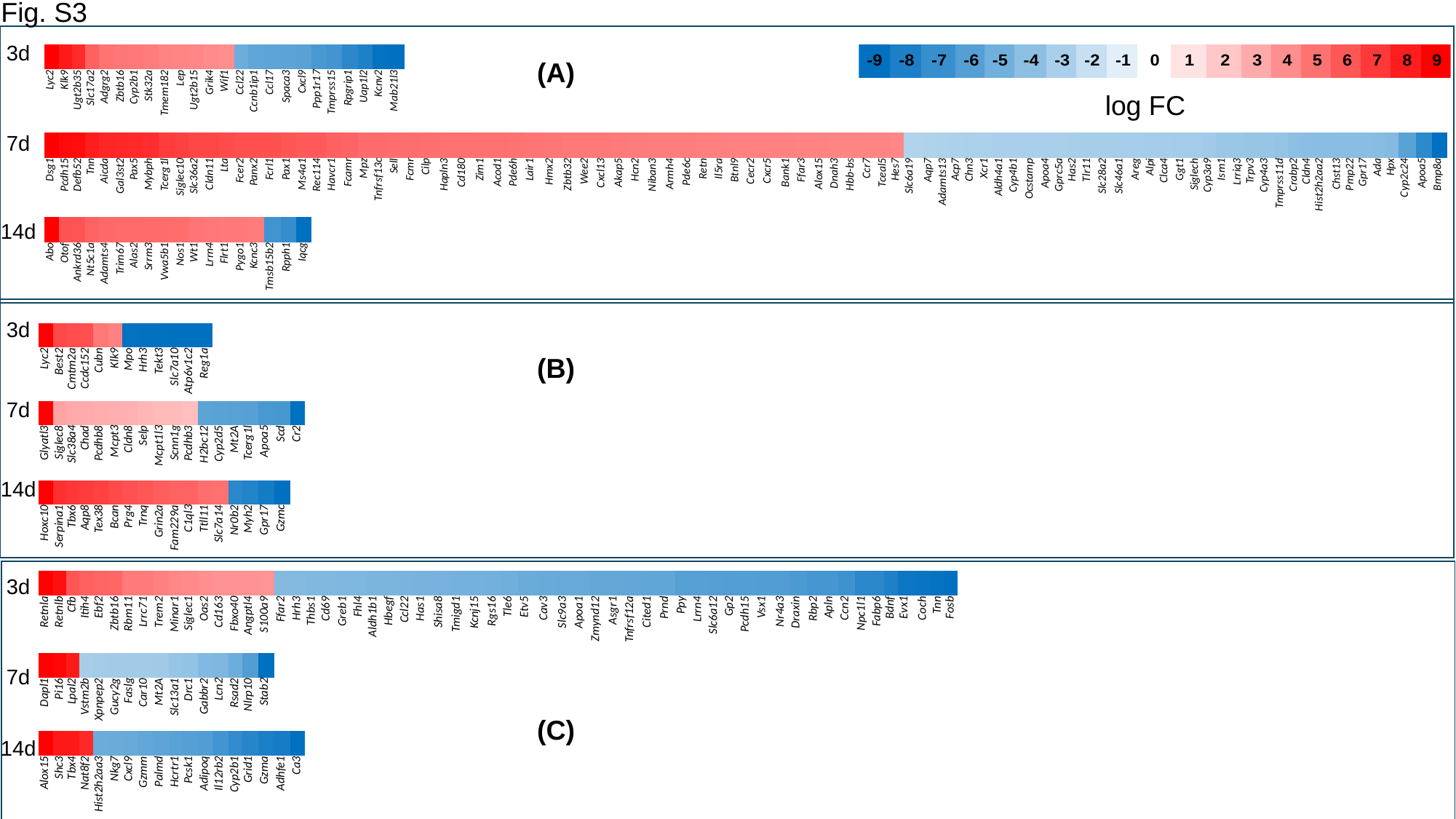

Fig. S3
3d
| | | | | | | | | | | | | | | | | | | | | | | | | | | | | | | | | | | | | | | | | | | | | | | | | | | | | | | | | | | | | | | | | | | | | | | | | | | | | | | | | | | | | | | | | | | |
| --- | --- | --- | --- | --- | --- | --- | --- | --- | --- | --- | --- | --- | --- | --- | --- | --- | --- | --- | --- | --- | --- | --- | --- | --- | --- | --- | --- | --- | --- | --- | --- | --- | --- | --- | --- | --- | --- | --- | --- | --- | --- | --- | --- | --- | --- | --- | --- | --- | --- | --- | --- | --- | --- | --- | --- | --- | --- | --- | --- | --- | --- | --- | --- | --- | --- | --- | --- | --- | --- | --- | --- | --- | --- | --- | --- | --- | --- | --- | --- | --- | --- | --- | --- | --- | --- | --- | --- | --- | --- | --- |
| Lyc2 | Klk9 | Ugt2b35 | Slc17a2 | Adgrg2 | Zbtb16 | Cyp2b1 | Stk32a | Tmem182 | Lep | Ugt2b15 | Grik4 | Wif1 | Ccl22 | Ccnb1ip1 | Ccl17 | Spaca3 | Cxcl9 | Ppp1r17 | Tmprss15 | Rpgrip1 | Uap1l2 | Kcnv2 | Mab21l3 | | | | | | | | | | | | | | | | | | | | | | | | | | | | | | | | | | | | | | | | | | | | | | | | | | | | | | | | | | | | | | | | | | | |
| | | | | | | | | | | | | | | | | | | | | | | | | | | | | | | | | | | | | | | | | | | | | | | | | | | | | | | | | | | | | | | | | | | | | | | | | | | | | | | | | | | | | | | | | | | |
| Dsg1 | Pcdh15 | Defb52 | Tnn | Aicda | Gal3st2 | Pax5 | Mybph | Tcerg1l | Siglec10 | Slc36a2 | Cldn11 | Lta | Fcer2 | Panx2 | Fcrl1 | Pax1 | Ms4a1 | Rec114 | Havcr1 | Fcamr | Mpz | Tnfrsf13c | Sell | Fcmr | Cilp | Hapln3 | Cd180 | Zim1 | Acod1 | Pde6h | Lair1 | Hmx2 | Zbtb32 | Wee2 | Cxcl13 | Akap5 | Hcn2 | Niban3 | Armh4 | Pde6c | Retn | Il5ra | Btnl9 | Cecr2 | Cxcr5 | Bank1 | Ffar3 | Alox15 | Dnah3 | Hbb-bs | Ccr7 | Tceal5 | Hes7 | Slc6a19 | Aqp7 | Adamts13 | Acp7 | Chn3 | Xcr1 | Aldh4a1 | Cyp4b1 | Ocstamp | Apoa4 | Gprc5a | Has2 | Tlr11 | Slc28a2 | Slc46a1 | Areg | Alpi | Clca4 | Ggt1 | Siglech | Cyp3a9 | Ism1 | Lrriq3 | Trpv3 | Cyp4a3 | Tmprss11d | Crabp2 | Cldn4 | Hist2h2aa2 | Chst13 | Pmp22 | Gpr17 | Ada | Hpx | Cyp2c24 | Apoa5 | Bmp8a |
| | | | | | | | | | | | | | | | | | | | | | | | | | | | | | | | | | | | | | | | | | | | | | | | | | | | | | | | | | | | | | | | | | | | | | | | | | | | | | | | | | | | | | | | | | | |
| Abo | Otof | Ankrd36 | Nt5c1a | Adamts4 | Trim67 | Alas2 | Srrm3 | Vwa5b1 | Nos1 | Wt1 | Lrrn4 | Flrt1 | Pygo1 | Kcnc3 | Tmsb15b2 | Rpph1 | Iqcg | | | | | | | | | | | | | | | | | | | | | | | | | | | | | | | | | | | | | | | | | | | | | | | | | | | | | | | | | | | | | | | | | | | | | | | | | |
(A)
log FC
7d
14d
3d
| | | | | | | | | | | | | | | | | | | | | | | | | | | | | | | | | | | | | | | | | | | | | | | | | | | | | | | | | | | | | | | | | | | | | | | | | | | | | | | | | | | | | | | | | | | |
| --- | --- | --- | --- | --- | --- | --- | --- | --- | --- | --- | --- | --- | --- | --- | --- | --- | --- | --- | --- | --- | --- | --- | --- | --- | --- | --- | --- | --- | --- | --- | --- | --- | --- | --- | --- | --- | --- | --- | --- | --- | --- | --- | --- | --- | --- | --- | --- | --- | --- | --- | --- | --- | --- | --- | --- | --- | --- | --- | --- | --- | --- | --- | --- | --- | --- | --- | --- | --- | --- | --- | --- | --- | --- | --- | --- | --- | --- | --- | --- | --- | --- | --- | --- | --- | --- | --- | --- | --- | --- | --- |
| Lyc2 | Best2 | Cmtm2a | Ccdc152 | Cubn | Klk9 | Mpo | Hrh3 | Tekt3 | Slc7a10 | Atp6v1c2 | Reg1a | | | | | | | | | | | | | | | | | | | | | | | | | | | | | | | | | | | | | | | | | | | | | | | | | | | | | | | | | | | | | | | | | | | | | | | | | | | | | | | |
| | | | | | | | | | | | | | | | | | | | | | | | | | | | | | | | | | | | | | | | | | | | | | | | | | | | | | | | | | | | | | | | | | | | | | | | | | | | | | | | | | | | | | | | | | | |
| Glyatl3 | Siglec8 | Slc38a4 | Chad | Pcdhb8 | Mcpt3 | Cldn8 | Selp | Mcpt1l3 | Scnn1g | Pcdhb3 | H2bc12 | Cyp2d5 | Mt2A | Tcerg1l | Apoa5 | Scd | Cr2 | | | | | | | | | | | | | | | | | | | | | | | | | | | | | | | | | | | | | | | | | | | | | | | | | | | | | | | | | | | | | | | | | | | | | | | | | |
| | | | | | | | | | | | | | | | | | | | | | | | | | | | | | | | | | | | | | | | | | | | | | | | | | | | | | | | | | | | | | | | | | | | | | | | | | | | | | | | | | | | | | | | | | | |
| Hoxc10 | Serpina1 | Tbx6 | Aqp8 | Tex38 | Bcan | Prg4 | Trnq | Grin2a | Fam229a | C1ql3 | Ttll11 | Slc7a14 | Nr0b2 | Myh2 | Gpr17 | Gzmc | | | | | | | | | | | | | | | | | | | | | | | | | | | | | | | | | | | | | | | | | | | | | | | | | | | | | | | | | | | | | | | | | | | | | | | | | | |
(B)
7d
14d
3d
| | | | | | | | | | | | | | | | | | | | | | | | | | | | | | | | | | | | | | | | | | | | | | | | | | | | | | | | | | | | | | | | | | | | | | | | | | | | | | | | | | | | | | | | | | | |
| --- | --- | --- | --- | --- | --- | --- | --- | --- | --- | --- | --- | --- | --- | --- | --- | --- | --- | --- | --- | --- | --- | --- | --- | --- | --- | --- | --- | --- | --- | --- | --- | --- | --- | --- | --- | --- | --- | --- | --- | --- | --- | --- | --- | --- | --- | --- | --- | --- | --- | --- | --- | --- | --- | --- | --- | --- | --- | --- | --- | --- | --- | --- | --- | --- | --- | --- | --- | --- | --- | --- | --- | --- | --- | --- | --- | --- | --- | --- | --- | --- | --- | --- | --- | --- | --- | --- | --- | --- | --- | --- |
| Retnla | Retnlb | Cfb | Itih4 | Ebf2 | Zbtb16 | Rbm11 | Lrrc71 | Trem2 | Minar1 | Siglec1 | Oas2 | Cd163 | Fbxo40 | Angptl4 | S100a9 | Ffar2 | Hrh3 | Thbs1 | Cd69 | Greb1 | Fhl4 | Aldh1b1 | Hbegf | Ccl22 | Has1 | Shisa8 | Tmigd1 | Kcnj15 | Rgs16 | Tle6 | Etv5 | Cav3 | Slc9a3 | Apoa1 | Zmynd12 | Asgr1 | Tnfrsf12a | Cited1 | Prnd | Ppy | Lrrn4 | Slc6a12 | Gp2 | Pcdh15 | Vsx1 | Nr4a3 | Draxin | Rbp2 | Apln | Ccn2 | Npc1l1 | Fabp6 | Bdnf | Evx1 | Coch | Tnn | Fosb | | | | | | | | | | | | | | | | | | | | | | | | | | | | | | | | | |
| | | | | | | | | | | | | | | | | | | | | | | | | | | | | | | | | | | | | | | | | | | | | | | | | | | | | | | | | | | | | | | | | | | | | | | | | | | | | | | | | | | | | | | | | | | |
| Dapl1 | Pi16 | Lpal2 | Vstm2b | Xpnpep2 | Gucy2g | Faslg | Car10 | Mt2A | Slc13a1 | Drc1 | Gabbr2 | Lcn2 | Rsad2 | Nlrp10 | Stab2 | | | | | | | | | | | | | | | | | | | | | | | | | | | | | | | | | | | | | | | | | | | | | | | | | | | | | | | | | | | | | | | | | | | | | | | | | | | |
| | | | | | | | | | | | | | | | | | | | | | | | | | | | | | | | | | | | | | | | | | | | | | | | | | | | | | | | | | | | | | | | | | | | | | | | | | | | | | | | | | | | | | | | | | | |
| Alox15 | Shc3 | Tbx4 | Nat8f2 | Hist2h2aa3 | Nkg7 | Cxcl9 | Gzmm | Palmd | Hcrtr1 | Pcsk1 | Adipoq | Il12rb2 | Cyp2b1 | Grid1 | Gzma | Adhfe1 | Ca3 | | | | | | | | | | | | | | | | | | | | | | | | | | | | | | | | | | | | | | | | | | | | | | | | | | | | | | | | | | | | | | | | | | | | | | | | | |
7d
(C)
14d
