# Supplementary material for: Evidence of an allostatic response by intestinal tissues following induction of joint inflammation
Source: PLoS One. 2026 Jan 23;21(1):e0338053. doi: 10.1371/journal.pone.0338053 (PMC12829947; doi:10.1371/journal.pone.0338053)
Supplement: S4 Fig — (PPTX) [file pone.0338053.s004.pptx]

## Slide 1
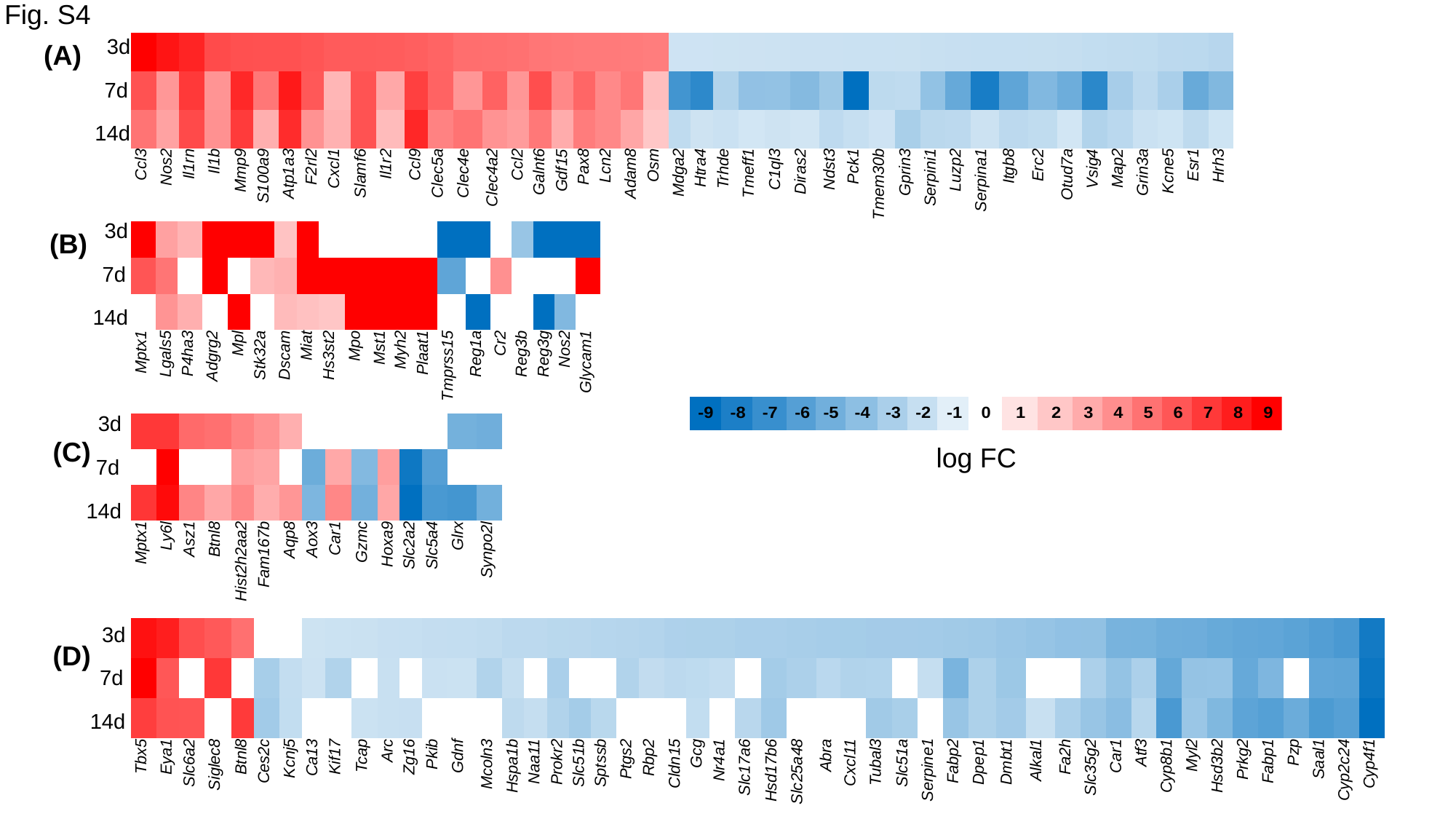

Fig. S4
3d
(A)
| | | | | | | | | | | | | | | | | | | | | | | | | | | | | | | | | | | | | | | | | | | | | | | | | | |
| --- | --- | --- | --- | --- | --- | --- | --- | --- | --- | --- | --- | --- | --- | --- | --- | --- | --- | --- | --- | --- | --- | --- | --- | --- | --- | --- | --- | --- | --- | --- | --- | --- | --- | --- | --- | --- | --- | --- | --- | --- | --- | --- | --- | --- | --- | --- | --- | --- | --- |
| | | | | | | | | | | | | | | | | | | | | | | | | | | | | | | | | | | | | | | | | | | | | | | | | | |
| | | | | | | | | | | | | | | | | | | | | | | | | | | | | | | | | | | | | | | | | | | | | | | | | | |
| Ccl3 | Nos2 | Il1rn | Il1b | Mmp9 | S100a9 | Atp1a3 | F2rl2 | Cxcl1 | Slamf6 | Il1r2 | Ccl9 | Clec5a | Clec4e | Clec4a2 | Ccl2 | Galnt6 | Gdf15 | Pax8 | Lcn2 | Adam8 | Osm | Mdga2 | Htra4 | Trhde | Tmeff1 | C1ql3 | Diras2 | Ndst3 | Pck1 | Tmem30b | Gprin3 | Serpini1 | Luzp2 | Serpina1 | Itgb8 | Erc2 | Otud7a | Vsig4 | Map2 | Grin3a | Kcne5 | Esr1 | Hrh3 | | | | | | |
7d
14d
3d
(B)
| | | | | | | | | | | | | | | | | | | | | | | | | | | | | | | | | | | | | | | | | | | | | | | | | | |
| --- | --- | --- | --- | --- | --- | --- | --- | --- | --- | --- | --- | --- | --- | --- | --- | --- | --- | --- | --- | --- | --- | --- | --- | --- | --- | --- | --- | --- | --- | --- | --- | --- | --- | --- | --- | --- | --- | --- | --- | --- | --- | --- | --- | --- | --- | --- | --- | --- | --- |
| | | | | | | | | | | | | | | | | | | | | | | | | | | | | | | | | | | | | | | | | | | | | | | | | | |
| | | | | | | | | | | | | | | | | | | | | | | | | | | | | | | | | | | | | | | | | | | | | | | | | | |
| Mptx1 | Lgals5 | P4ha3 | Adgrg2 | Mpl | Stk32a | Dscam | Miat | Hs3st2 | Mpo | Mst1 | Myh2 | Plaat1 | Tmprss15 | Reg1a | Cr2 | Reg3b | Reg3g | Nos2 | Glycam1 | | | | | | | | | | | | | | | | | | | | | | | | | | | | | | |
7d
14d
3d
| | | | | | | | | | | | | | | | | | | | | | | | | | | | | | | | | | | | | | | | | | | | | | | | | | |
| --- | --- | --- | --- | --- | --- | --- | --- | --- | --- | --- | --- | --- | --- | --- | --- | --- | --- | --- | --- | --- | --- | --- | --- | --- | --- | --- | --- | --- | --- | --- | --- | --- | --- | --- | --- | --- | --- | --- | --- | --- | --- | --- | --- | --- | --- | --- | --- | --- | --- |
| | | | | | | | | | | | | | | | | | | | | | | | | | | | | | | | | | | | | | | | | | | | | | | | | | |
| | | | | | | | | | | | | | | | | | | | | | | | | | | | | | | | | | | | | | | | | | | | | | | | | | |
| Mptx1 | Ly6l | Asz1 | Btnl8 | Hist2h2aa2 | Fam167b | Aqp8 | Aox3 | Car1 | Gzmc | Hoxa9 | Slc2a2 | Slc5a4 | Glrx | Synpo2l | | | | | | | | | | | | | | | | | | | | | | | | | | | | | | | | | | | |
(C)
log FC
7d
14d
3d
| | | | | | | | | | | | | | | | | | | | | | | | | | | | | | | | | | | | | | | | | | | | | | | | | | |
| --- | --- | --- | --- | --- | --- | --- | --- | --- | --- | --- | --- | --- | --- | --- | --- | --- | --- | --- | --- | --- | --- | --- | --- | --- | --- | --- | --- | --- | --- | --- | --- | --- | --- | --- | --- | --- | --- | --- | --- | --- | --- | --- | --- | --- | --- | --- | --- | --- | --- |
| | | | | | | | | | | | | | | | | | | | | | | | | | | | | | | | | | | | | | | | | | | | | | | | | | |
| | | | | | | | | | | | | | | | | | | | | | | | | | | | | | | | | | | | | | | | | | | | | | | | | | |
| Tbx5 | Eya1 | Slc6a2 | Siglec8 | Btnl8 | Ces2c | Kcnj5 | Ca13 | Kif17 | Tcap | Arc | Zg16 | Pkib | Gdnf | Mcoln3 | Hspa1b | Naa11 | Prokr2 | Slc51b | Sptssb | Ptgs2 | Rbp2 | Cldn15 | Gcg | Nr4a1 | Slc17a6 | Hsd17b6 | Slc25a48 | Abra | Cxcl11 | Tubal3 | Slc51a | Serpine1 | Fabp2 | Dpep1 | Dmbt1 | Alkal1 | Fa2h | Slc35g2 | Car1 | Atf3 | Cyp8b1 | Myl2 | Hsd3b2 | Prkg2 | Fabp1 | Pzp | Saal1 | Cyp2c24 | Cyp4f1 |
(D)
7d
14d
