# Supplementary figures and images for: Evidence of an allostatic response by intestinal tissues following induction of joint inflammation
Source: PLoS One. 2026 Jan 23;21(1):e0338053. doi: 10.1371/journal.pone.0338053 (PMC12829947; doi:10.1371/journal.pone.0338053)

## Slide 1
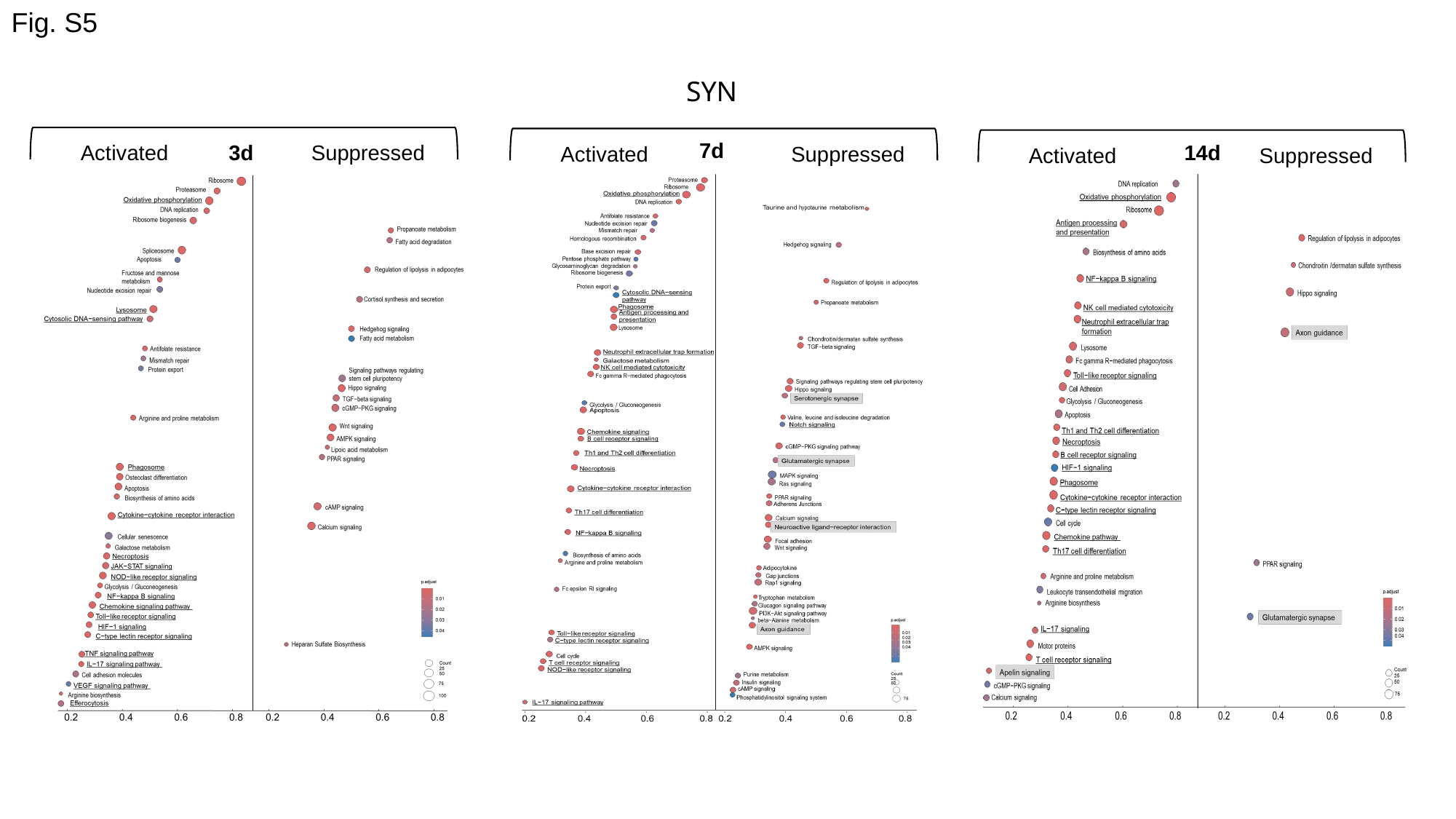

Fig. S5
SYN
7d
14d
Activated
3d
Suppressed
Activated
Suppressed
Activated
Suppressed

Supplement: S5 Fig — Pathways (ranked by p-value) were assigned to color-coded functional categories as listed in the KEGG Database (Metabolism, Cellular Processes, Genetic Information Processing, Environmental Processing, Immune System, Nervous System). Gene ratio for a given pathway is computed as the percentage of genes present divided by the total number of genes in that pathway. Activated and Suppressed pathways are separated by a solid black vertical line and gene ratios 0.5 are separated by a dotted vertical line. The top 20 modified pathways are above the horizontal dotted line. (PPTX) [file pone.0338053.s005.pptx]

## Slide 1
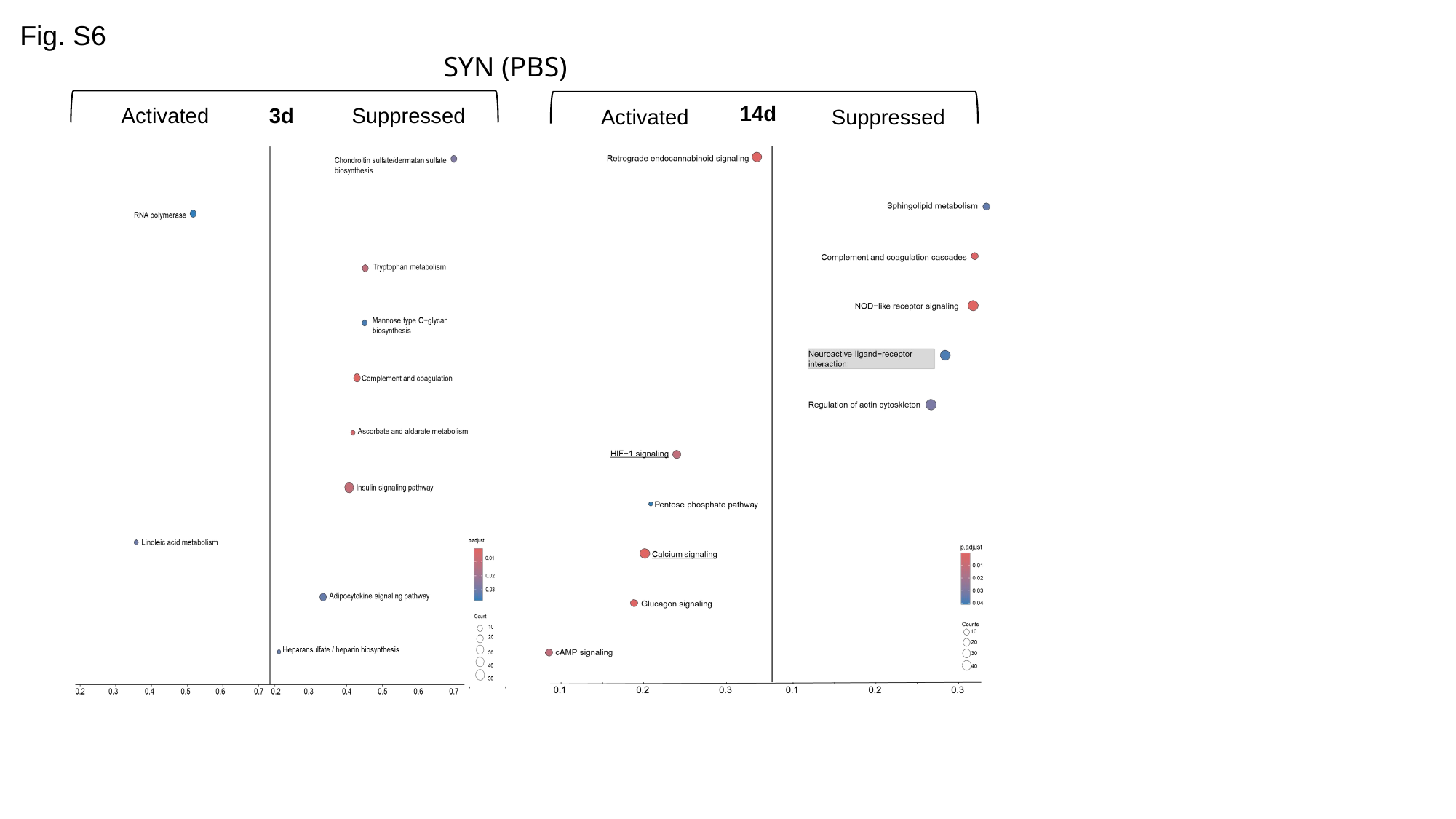

Fig. S6
SYN (PBS)
14d
Activated
3d
Suppressed
Activated
Suppressed

Supplement: S6 Fig — Pathways (ranked by p-value) were assigned to color-coded functional categories as listed in the KEGG Database (Metabolism, Cellular Processes, Genetic Information Processing, Environmental Processing, Immune System, Nervous System). Gene ratio for a given pathway is computed as the percentage of genes present divided by the total number of genes in that pathway. Activated and Suppressed pathways are separated by a solid black vertical line and gene ratios 0.5 are separated by a dotted vertical line. The top 20 modified pathways are above the horizontal dotted line. (PPTX) [file pone.0338053.s006.pptx]

## Slide 1
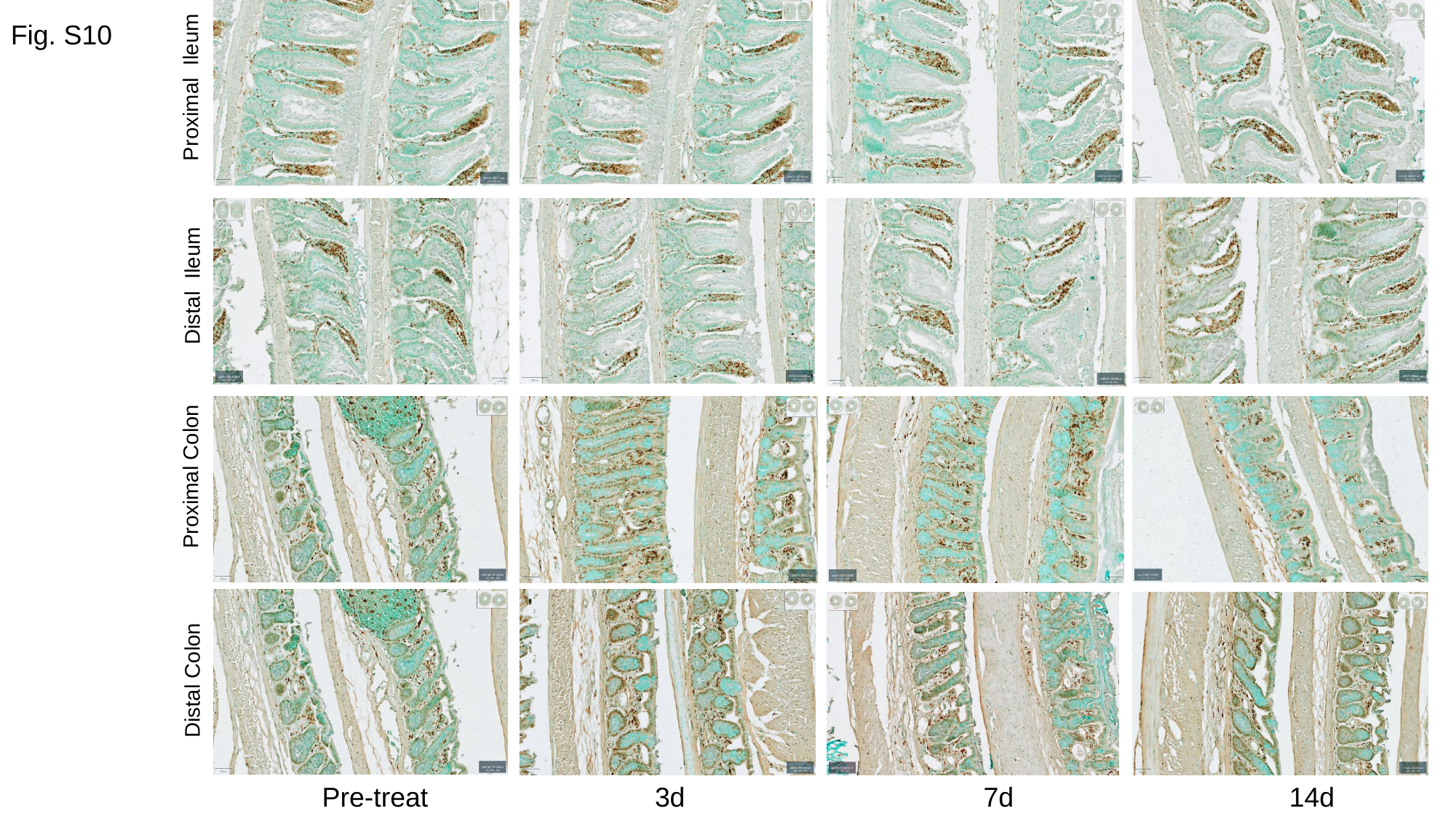

Fig. S10
Proximal Ileum
Distal Ileum
Proximal Colon
Distal Colon
Pre-treat
3d
7d
14d

Supplement: S10 Fig — Images represent regions from the scanned slides that were used for panels shown in Fig 10. (PPTX) [file pone.0338053.s010.pptx]

## Slide 1
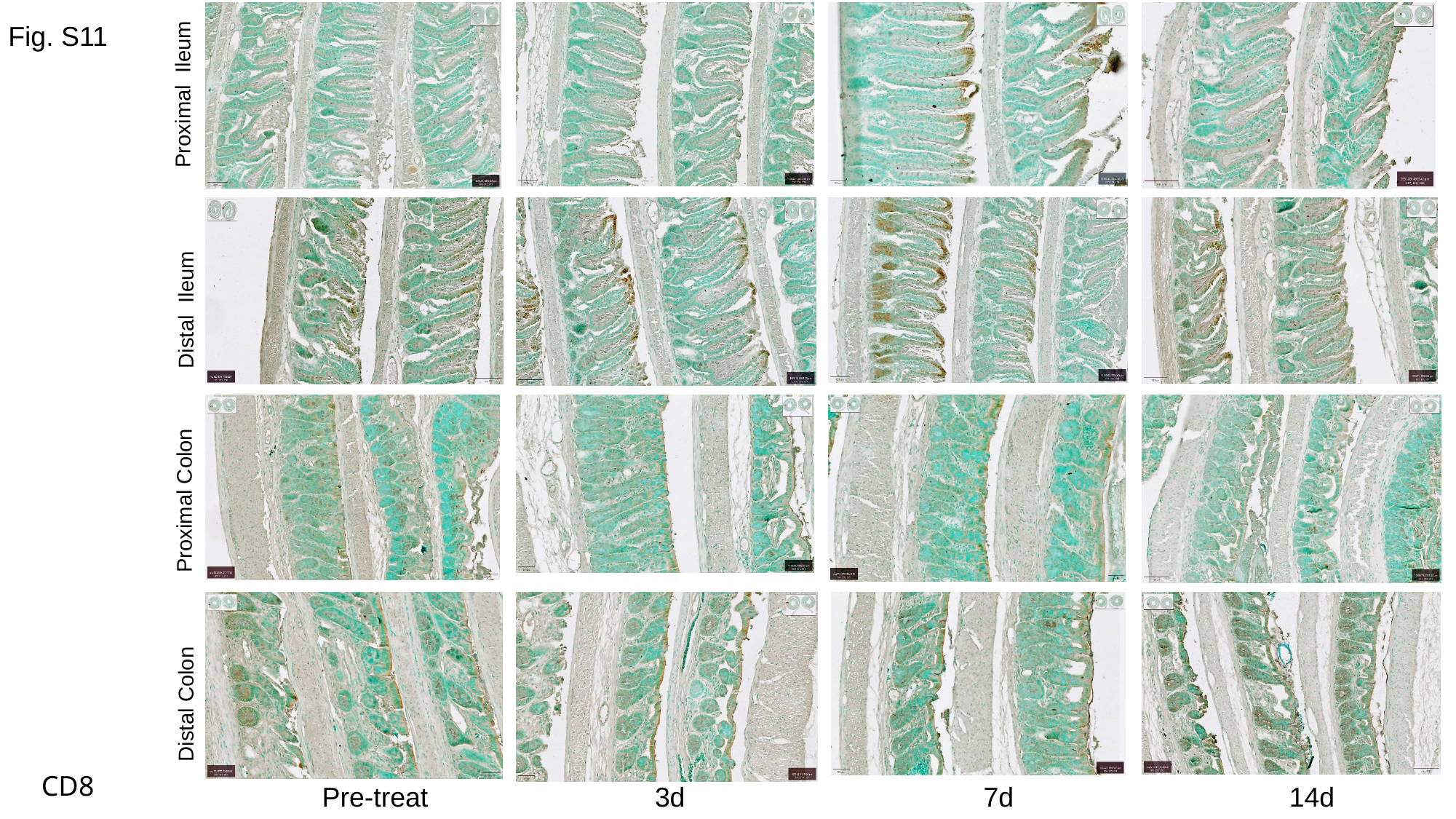

Fig. S11
Proximal Ileum
Distal Ileum
Proximal Colon
Distal Colon
CD8
Pre-treat
3d
7d
14d

Supplement: S11 Fig — Images represent regions from the scanned slides that were used for panels shown in Fig 10. (PPTX) [file pone.0338053.s011.pptx]

## Slide 1
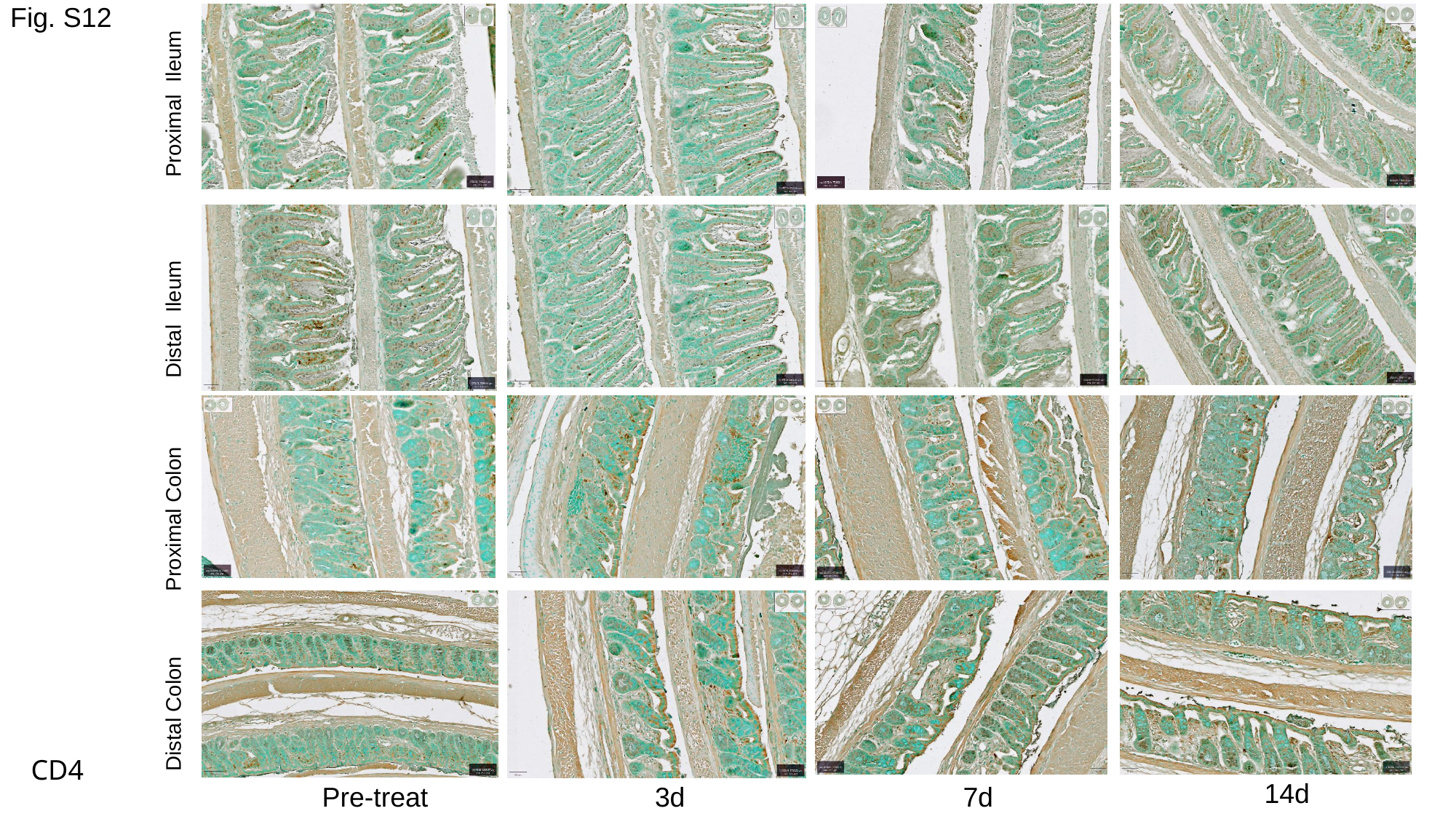

Fig. S12
Proximal Ileum
Distal Ileum
Proximal Colon
Distal Colon
CD4
14d
Pre-treat
3d
7d

Supplement: S12 Fig — Images represent regions from the scanned slides that were used for panels shown in Fig 10. (PPTX) [file pone.0338053.s012.pptx]

## Slide 1
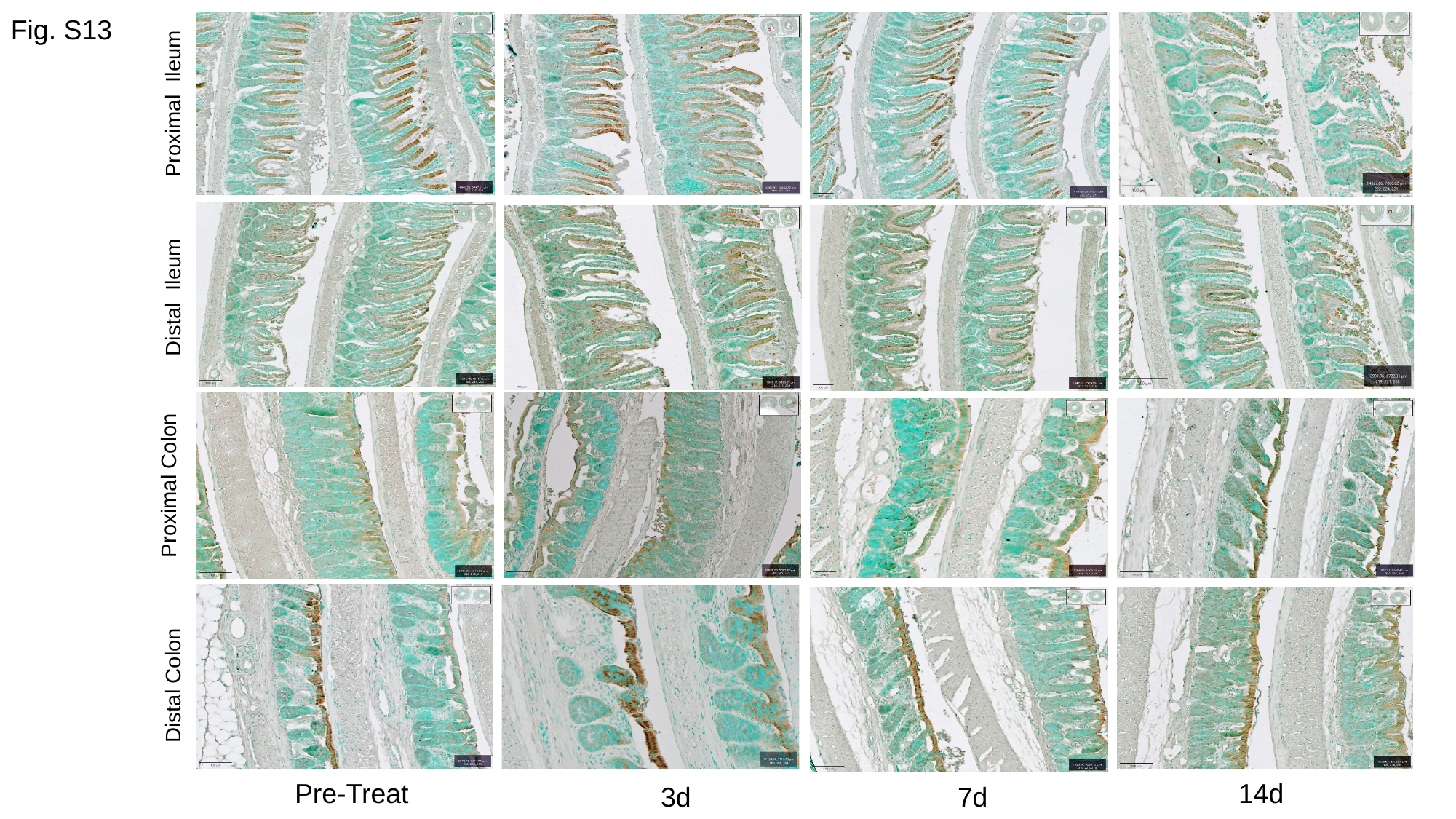

Fig. S13
Proximal Ileum
Distal Ileum
Proximal Colon
Distal Colon
Pre-Treat
14d
3d
7d

Supplement: S13 Fig — Images represent regions from the scanned slides that were used for panels shown in Fig 10. (PPTX) [file pone.0338053.s013.pptx]

## Slide 1
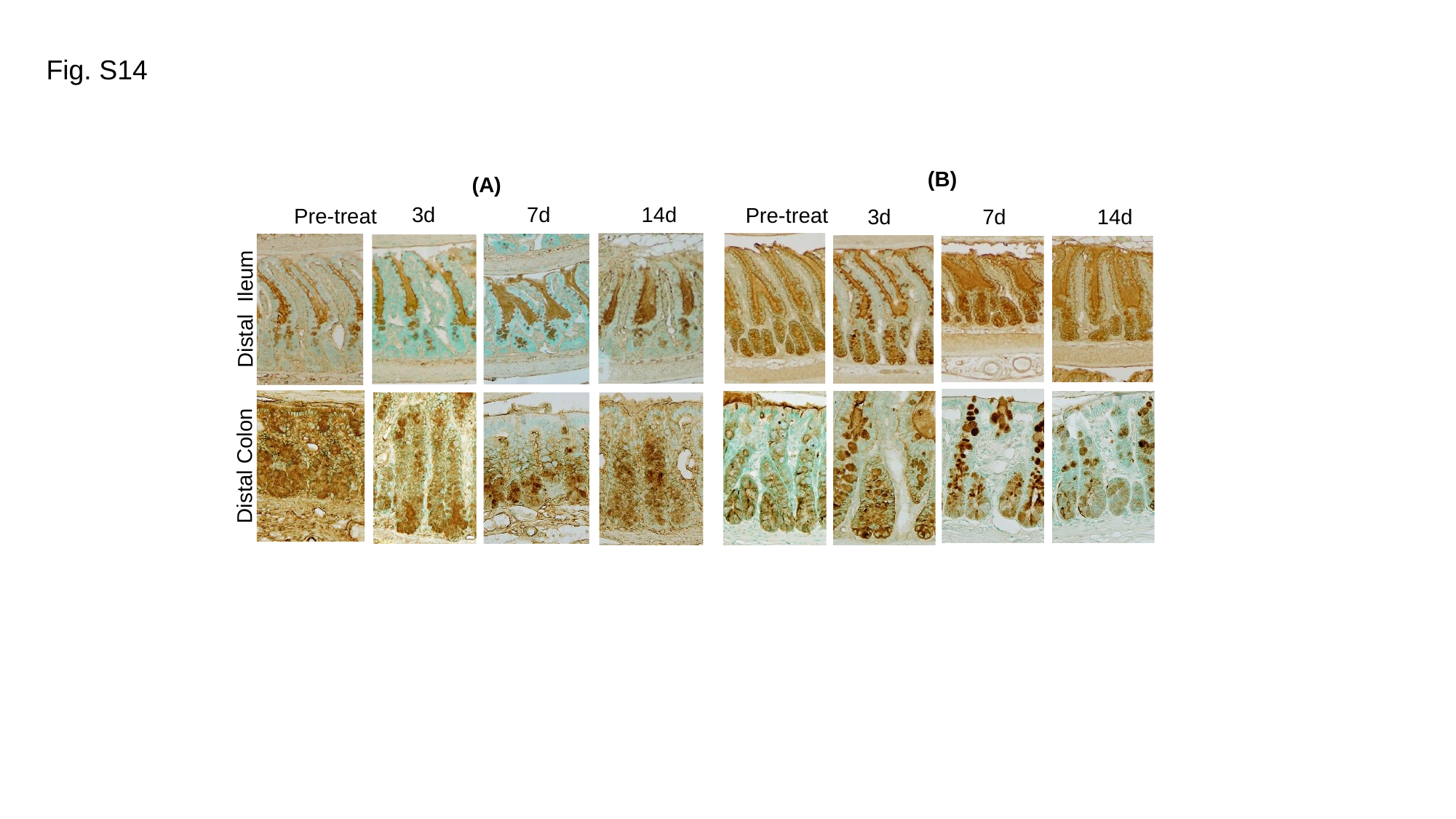

Fig. S14
(B)
(A)
3d
7d
14d
Pre-treat
Pre-treat
3d
7d
14d
Distal Ileum
Distal Colon

Supplement: S14 Fig — Images represent regions from the scanned slides that were used for panels shown in Fig. 10. Mal II (A) and UEA (B). (PPTX) [file pone.0338053.s014.pptx]

## Slide 1
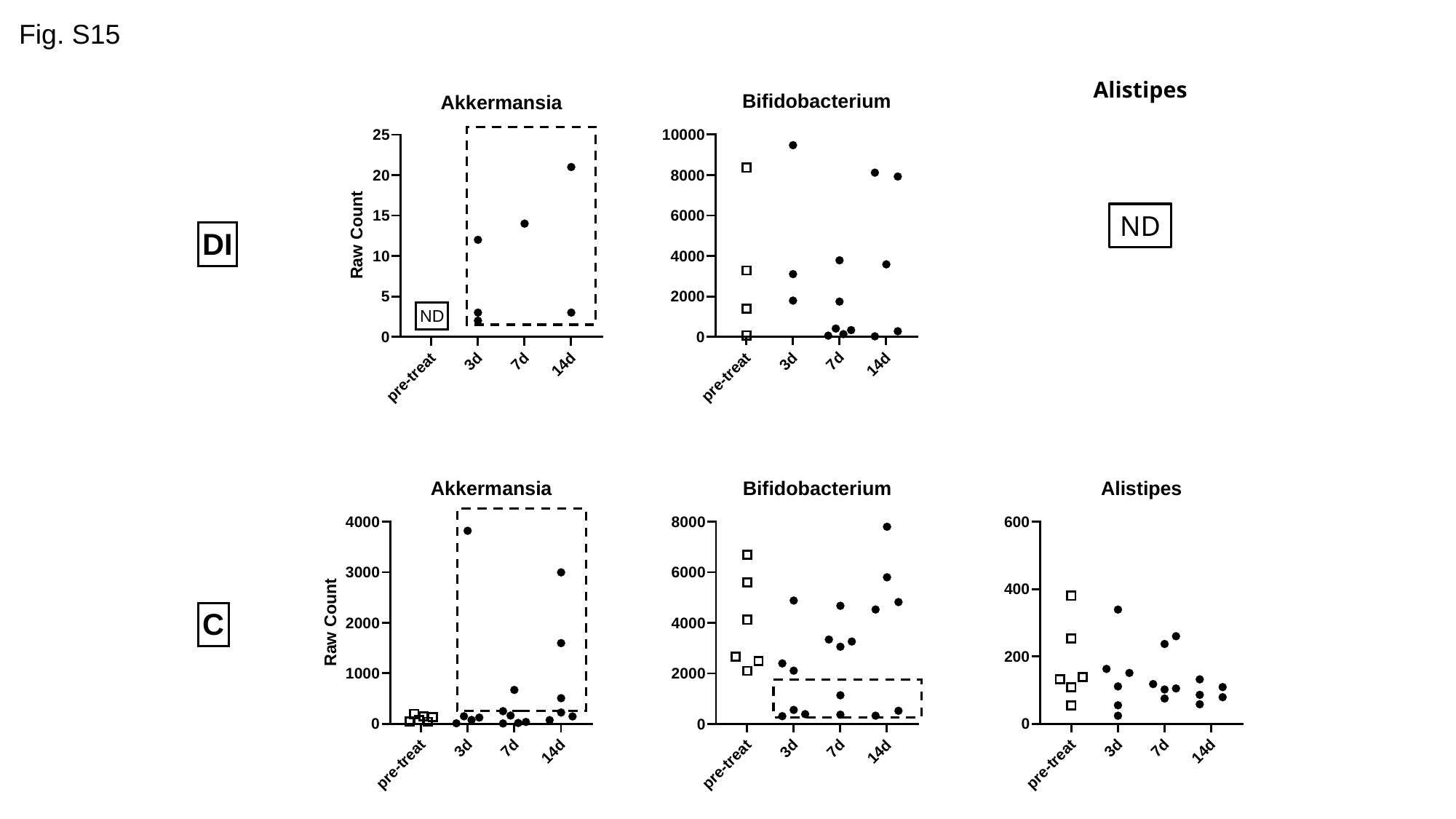

Fig. S15
Alistipes
ND

Supplement: S15 Fig — Raw counts for mucin-degrading bacteria (Akkermansia, Alistipes) and sialic acid cleaving bacteria (Bifidobacterium) are shown. Note that in pre-treatment rats, Akkermansia and Alistipes were below detection in the ileum (ND = not detected). Variable animal-to-animal responses are outlined in dotted lines. Note the much lower counts for Akkermansia and Alistipes in the ileum compared to the colon. Bifidobacterium showed higher counts in both intestinal regions. (PPTX) [file pone.0338053.s015.pptx]
