# Supplementary material for: Evidence of an allostatic response by intestinal tissues following induction of joint inflammation
Source: PLoS One. 2026 Jan 23;21(1):e0338053. doi: 10.1371/journal.pone.0338053 (PMC12829947; doi:10.1371/journal.pone.0338053)
Supplement: S7 Fig — Pathways (ranked by p-value) were assigned to color-coded functional categories as listed in the KEGG Database (Metabolism, Cellular Processes, Genetic Information Processing, Environmental Processing, Immune System, Nervous System). Gene ratio for a given pathway is computed as the percentage of genes present divided by the total number of genes in that pathway. Activated and Suppressed pathways are separated by a solid black vertical line and gene ratios 0.5 are separated by a dotted vertical line. The top 20 modified pathways are indicated by a dotted vertical line. (PPTX) [file pone.0338053.s007.pptx]

## Slide 1
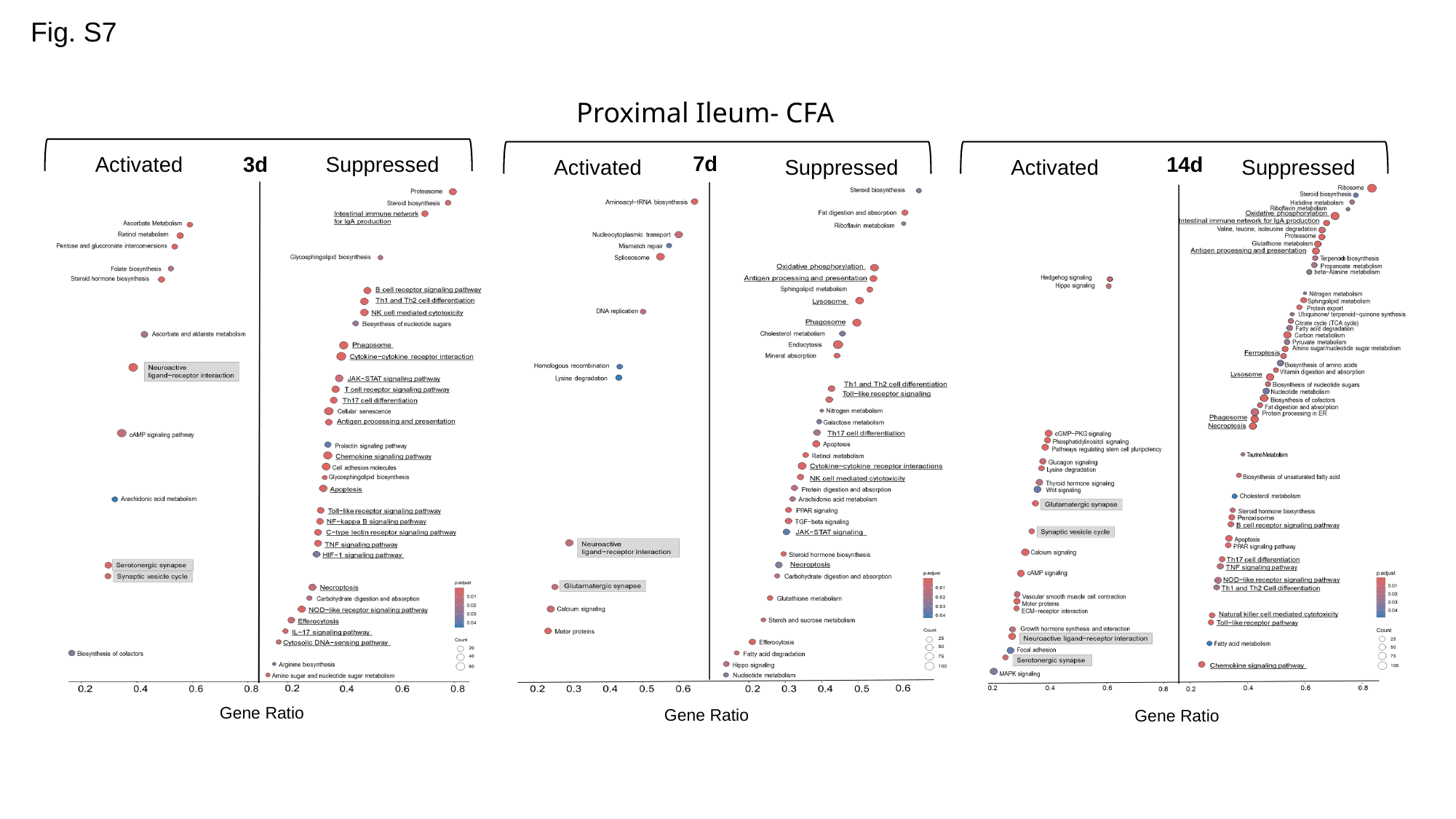

Fig. S7
Proximal Ileum- CFA
7d
14d
Activated
3d
Suppressed
Activated
Suppressed
Activated
Suppressed
Gene Ratio
Gene Ratio
Gene Ratio
