# Supplementary material for: Evidence of an allostatic response by intestinal tissues following induction of joint inflammation
Source: PLoS One. 2026 Jan 23;21(1):e0338053. doi: 10.1371/journal.pone.0338053 (PMC12829947; doi:10.1371/journal.pone.0338053)
Supplement: S8 Fig — Activated pathways are listed on the left and suppressed pathways on the right. Gene ratios and pathway groupings are assigned as described for S6 Fig. (PPTX) [file pone.0338053.s008.pptx]

## Slide 1
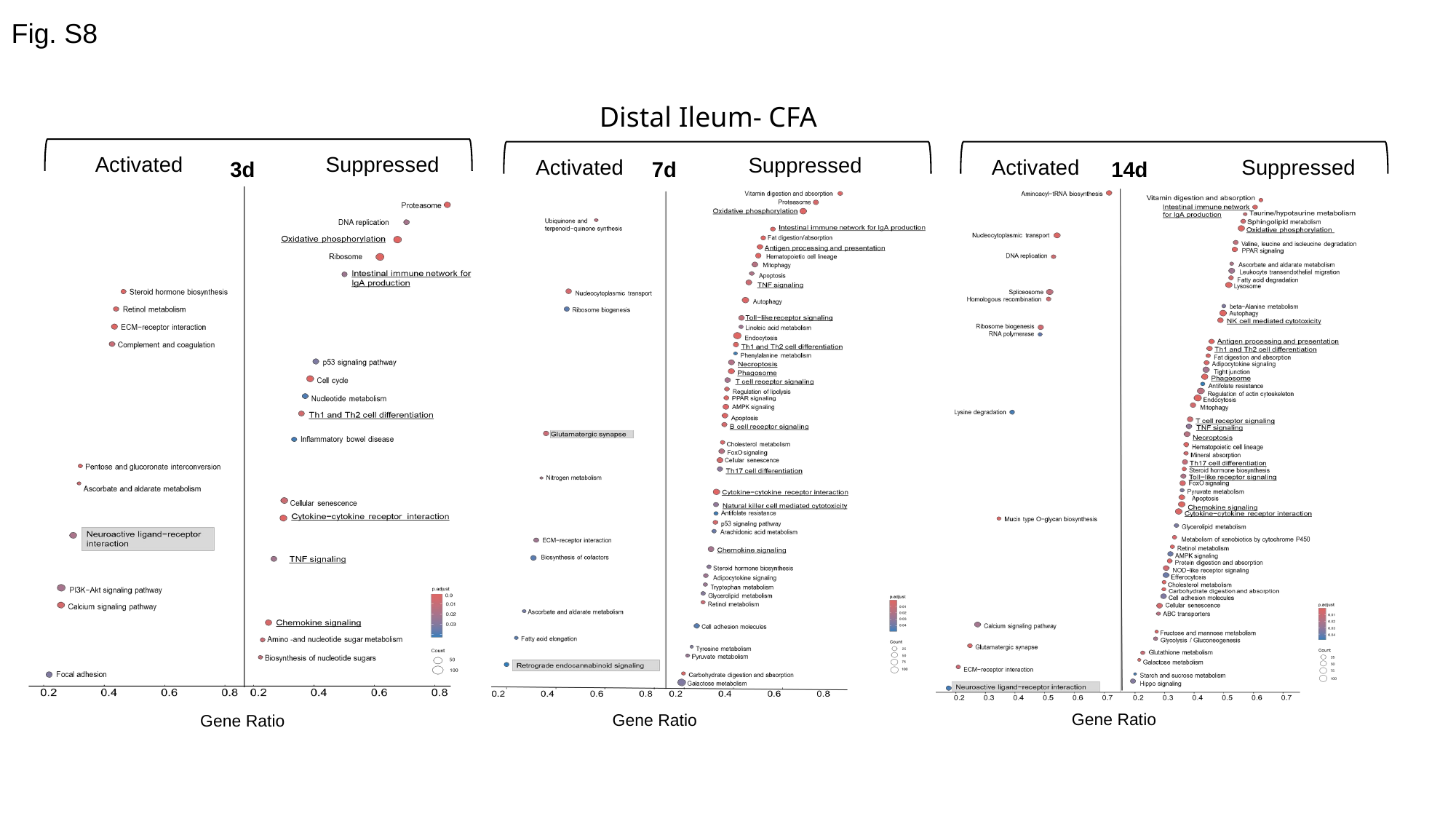

Fig. S8
Distal Ileum- CFA
Activated
Suppressed
Suppressed
Activated
Activated
Suppressed
3d
7d
14d
Gene Ratio
Gene Ratio
Gene Ratio
