# Supplementary material for: Evidence of an allostatic response by intestinal tissues following induction of joint inflammation
Source: PLoS One. 2026 Jan 23;21(1):e0338053. doi: 10.1371/journal.pone.0338053 (PMC12829947; doi:10.1371/journal.pone.0338053)
Supplement: Attachment — Submitted filename: Response to Reviewers_24Oct25.docx [file pone.0338053.s017.docx]

**Journal Requirements:**

When submitting your revision, we need you to address these additional requirements.

1. Please ensure that your manuscript meets PLOS ONE's style requirements, including those for file naming. The PLOS ONE style templates can be found at <https://journals.plos.org/plosone/s/file?id=wjVg/PLOSOne_formatting_sample_main_body.pdf> and <https://journals.plos.org/plosone/s/file?id=ba62/PLOSOne_formatting_sample_title_authors_affiliations.pdf>.

2.  PLOS ONE now requires that authors provide the original uncropped and unadjusted images underlying all blot or gel results reported in a submission’s figures or Supporting Information files. This policy and the journal’s other requirements for blot/gel reporting and figure preparation are described in detail at <https://journals.plos.org/plosone/s/figures#loc-blot-and-gel-reporting-requirements> and <https://journals.plos.org/plosone/s/figures#loc-preparing-figures-from-image-files>. When you submit your revised manuscript, please ensure that your figures adhere fully to these guidelines and provide the original underlying images for all blot or gel data reported in your submission. See the following link for instructions on providing the original image data: <https://journals.plos.org/plosone/s/figures#loc-original-images-for-blots-and-gels>.

In your cover letter, please note whether your blot/gel image data are in Supporting Information or posted at a public data repository, provide the repository URL if relevant, and provide specific details as to which raw blot/gel images, if any, are not available. Email us at [plosone@plos.org](mailto:plosone@plos.org) if you have any questions.

- **Response:** The blots shown in the Supporting Information Figure S2 contain full, unadjusted and uncropped gels.

3.  Thank you for stating the following in the Acknowledgments Section of your manuscript:

[We would like to thank Dr. Georgia Papavasiliou (Illinois Institute of Technology) and Dr. Joseph Reynold (Rosalind Franklin University) for their insightful discussions on intestinal immunology and therapeutic target discovery during the progression of this project; Dr. Stefan Green for his input in study design and genomic analyses; and Rylan Martin for technical support. Funding for this project is from the Orthopedics Departmental Fund RUMC (MMM), the Katz/Rubschlager Endowed Chair at RUMC (AP), and Versus Arthritis, UK Grant 22277 (AJD and CMM).]

We note that you have provided funding information that is not currently declared in your Funding Statement. However, funding information should not appear in the Acknowledgments section or other areas of your manuscript. We will only publish funding information present in the Funding Statement section of the online submission form. Please remove any funding-related text from the manuscript and let us know how you would like to update your Funding Statement. Currently, your Funding Statement reads as follows: [Funding for this project is from the Orthopedics Departmental Fund RUMC, no grant number (MMM), the Katz/Rubschlager Endowed Chair at RUMC (AP), and Versus Arthritis, UK Grant 22277 (AJD and CMM). None of the sponsors or funders had any role in the study design, data collection and analysis, decision to publish, or preparation of the manuscript.]

Please include your amended statements within your cover letter; we will change the online submission form on your behalf.

- **Response:** Thanks- we have removed the funding information from the acknowledgements and added this text to our cover letter for it to be added by PlosOne to the appropriate form.

4. When completing the data availability statement of the submission form, you indicated that you will make your data available on acceptance. We strongly recommend all authors decide on a data sharing plan before acceptance, as the process can be lengthy and hold up publication timelines. Please note that, though access restrictions are acceptable now, your entire data will need to be made freely accessible if your manuscript is accepted for publication. This policy applies to all data except where public deposition would breach compliance with the protocol approved by your research ethics board. If you are unable to adhere to our open data policy, please kindly revise your statement to explain your reasoning and we will seek the editor's input on an exemption. Please be assured that, once you have provided your new statement, the assessment of your exemption will not hold up the peer review process.

- **Response:** Thank you for this reminder. All sequencing reads generated in this study have been deposited in the National Center for Biotechnology Information (NCBI) BioProject database under accession numbers PRJNA 1347841 and 1348463 (RNASeq), PRJNA 1348465 and 1348470 (Microbiota). This statement has been added to the data sharing and availability statement.

5. We note that you have included the phrase “data not shown” in your manuscript. Unfortunately, this does not meet our data sharing requirements. PLOS does not permit references to inaccessible data. We require that authors provide all relevant data within the paper, Supporting Information files, or in an acceptable, public repository. Please add a citation to support this phrase or upload the data that corresponds with these findings to a stable repository (such as Figshare or Dryad) and provide URLs, DOIs, or accession numbers that may be used to access these data. Or, if the data are not a core part of the research being presented in your study, we ask that you remove the phrase that refers to these data.

- **Response:** Thank you for this comment. We have removed the ‘data not shown’ and added the fold values +/- SD to the Discussion text pg. 14: “Generation of such a matrix in joint tissues is likely involved in the acute phase of this model, since RNASeq data show a 1.8 +/- 0.12 and 2.7 +/- 0.11 fold increase in mRNA levels for TSG6 (Tnfaip6) and hyaluronan synthase 2 (Has2), respectively at 3d post CFA, with both genes returning to pre-inflammation levels by 7d”.

6. Please include your full ethics statement in the ‘Methods’ section of your manuscript file. In your statement, please include the full name of the IRB or ethics committee who approved or waived your study, as well as whether or not you obtained informed written or verbal consent. If consent was waived for your study, please include this information in your statement as well.

- **Response:** Our study uses a preclinical model and not human subjects or tissues therefore no IRB is required to be reported. We have included ethical treatment of animals protocol number to the methods section.

7. Please include captions for your Supporting Information files at the end of your manuscript, and update any in-text citations to match accordingly. Please see our Supporting Information guidelines for more information: <http://journals.plos.org/plosone/s/supporting-information>.

- **Response:** We have added the supporting information figure legend at the end of the manuscript.

8. We notice that your supplementary figures are uploaded with the file type 'Figure'. Please amend the file type to 'Supporting Information'. Please ensure that each Supporting Information file has a legend listed in the manuscript after the references list.

- **Response:** Thank you for this comment. We have added the supporting information figure legend at the end of the manuscript and uploaded the supplementary figures as Supporting Information Figures.

9. If the reviewer comments include a recommendation to cite specific previously published works, please review and evaluate these publications to determine whether they are relevant and should be cited. There is no requirement to cite these works unless the editor has indicated otherwise.

- **Response:** Thank you. We have included citations as suggested by reviewers, now cited in the text and added to the reference list.

- We thank the reviewers for their careful reading of the manuscript and their helpful comments. We have addressed each of the queries below and modifications are shown in the manuscript using track changes.

**5. Review Comments to the Author**

Please use the space provided to explain your answers to the questions above. You may also include additional comments for the author, including concerns about dual publication, research ethics, or publication ethics. (Please upload your review as an attachment if it exceeds 20,000 characters)

**Reviewer #1:** The manuscript by Meghan Moran and colleagues entitled "Evidence of an Allostatic Response by Intestinal Tissues Following Induction of Joint Inflammation", presents an interesting and well-conducted study. While the writing is generally good, certain sections would benefit from improved cohesion and clarity. My minor comments are as follows:

**Query 1**. Include relevant statistical data on the disease burden of gut health issues linked to arthritis, bone disorders, or the joint–gut communication axis.

**Response:** Thanks for this comment. We have edited and added new text and additional citations that now highlight statistical data on GI symptoms in RA and other bone diseases in the Introduction, 1^st^ paragraph.

**Query 2.** Consider adding, either in the introduction or discussion, how metabolic-inflammatory disorders are associated with arthritis—particularly osteoarthritis as shown in recent studies (PMID: 34537381).

**Response**: Thank you for drawing attention to this reference. Whilst it does not directly address the effect of joint inflammation on intestinal health, we have added it in the Introduction Page 3: *(REF PMID: 34537381)* to draw attention to the possible effect of metabolic disorders on progression of OA due their effect on gut health.

**Query 3.** In the discussion: the statement "Transmission of signals between diseased tissues and the gut", clarify what is meant by diseased tissues.

**Response**: We have modified the text on Page 16 in the Discussion and added pertinent new references: *“ Transmission of signals between diseased tissues such as bone, kidney (PMID: 31560235), heart (PMID: 40403109), liver (PMID: 38280375) and brain (PMID: 34822299) and the gut has been reported for a wide range of disease conditions, but the molecular and cell biological mechanisms remain largely undefined, except for the much-studied bidirectional pathway between the brain and the intestine [142], The later includes cytokine, chemokines, migrating or circulating immune cells, and microbiota-derived metabolites. More relevant as a potential mediator of intestinal responses to joint inflammation is the enteric nervous system (ENS), which is a highly conserved network of neuron and glial cells located throughout the intestinal tract [143].”*

**Query 4.** For methods section, provide detailed catalogue numbers, clone numbers for antibodies, and company names for all kits and reagents used.

**Response:** Thank you for this comment. We have added the company and catalogue information to the Methods Section, pg. 18.

**Query 5**. Clarify whether the bulk RNA-seq analyses of synovium and intestine were generated from age-matched animals.

**Response**: We have added a statement to the Method Section Page 19: *“All tissues were collected from sex and age matched rats”.*

**Query 6.** Expand on the synovial fluid collection procedure.

**Response:** We have clarified the method description on page 13: *“To retrieve synovial fluid the joint cavity was rinsed twice with 150µL 1xPBS and collected into sterile microcentrifuge tubes. Lavages from right and left knee joints from each individual rat were combined and centrifuged at 3,000 rpm/805 g for 10 min at 4°C to remove cells and debris. Supernatants were stored at -80ºC until further analyses.”*

**Query 7**. Add missing p-values to most of the statistical data in Figure 1 panels.

**Response:** Thank you for this comment. The p-values are now represented in the graphs of Fig. 1 using * and a key that is in the legend.

**Query 8.** For Figures 4 to 6, consider including IPA or GO pathway analyses to identify cell-specific immune signaling pathways for myeloid and lymphoid populations (not just broad terminology) alongside the volcano plots in the same figure panel ---

**Response:** Thank you for this comment. For pathway analysis, we did indeed run GSEA against both KEGG database and the msigDB database. We used the results from KEGG in the manuscript as they are curated and named in a more understandable terminology. We do have results of pathway analysis GSEA against the msigDB Gene Ontology gene sets (subsets BP, MF, and CC). We added an additional table/figure view all the pathways and genes that were detected within the GO gene sets. We have added this as NEW FIGURE 9 with the Figure Legend “*New Figure 9*: Myeloid and Lymphoid-associated pathway analyses using GSEA using against the msigDB Gene Ontology gene sets, subset BP. Activated pathways are marked with a red upward pointing arrow (↑) and suppressed pathways with a blue downward pointing arrow (↓). No change is indicated by a horizontal double arrow (↔). SYN= synovium, PI = proximal ileum, DI = distal ileum, Co= Colon.”q

Also, the following text was added in the **Results Section pg. 11**: “Additional RNASeq data analyses to define modulation of Myeloid or Lymphoid-specific pathway using the GSEA data base against the msigDB Gene Ontology gene sets (subset BP) are shown in Fig 9. The majority of identified GOs, i.e. 22, were associated with cells from the myeloid lineage, 11 of those were for leukocytes and 5 for dendritic cells. All pathways, except for “Myeloid Development” in the distal colon, were downregulated relative to naïve intestinal regions. The number of affected pathways with decreased transcript abundance was largely in the proximal ileum, fewer in the distal colon, but largely unaffected in the distal ileum. Moreover, pathway modulations were most pronounced at 3d in all regions but largely restored to pre-injury levels by 14d post joint injury. Lymphoid pathways were minimally affected. “Lymphoid organ development” was suppressed in the proximal ileum and lymphoid progenitor cell differentiation activated at d14 in the distal colon.”

Lastly, we added text in the **Discussion Section pg.15-16**: “In addition, specific immune cell associated pathways (Fig. 9) in all three regions of the intestine, showed suppression of a broad range of immune-associated responses characteristic of resident leucocytes and dendritic cells, whereas lymphoid cell types were essentially unaffected  in response to the joint inflammation. Such broad “gene silencing” might be expected as a response to prevent chronic damage to the essential functions of the intestine, including nutrient digestion and absorption, as well as a steady-state defense against bacterial invasion to support microbial symbiosis that might be affected if an innate immune response is activated in the gut by inflammation in a “distant” organ.”

Reviewer #2: The manuscript “Evidence of an Allostatic Response by Intestinal Tissues Following Induction of Joint Inflammation” investigates the inter-organ communication between inflamed joint tissues and the intestinal barrier using a rat model of mono-joint inflammatory arthritis induced by Complete Freund’s Adjuvant (CFA). The authors explore how acute joint inflammation affects intestinal tissues at the molecular, cellular, and microbiome levels, proposing an allostatic response—a concept from stress physiology—within the gut.
The text is well writing and clear, so I have few concerns about it, as explicated below:

**Query 1:** The title brings the term joint inflammation, but the central topic of the manuscript is an experimental rheumatoid arthritis. Can the authors explain the choice of the term in the title?

**Response:** On Page 4, we have clarified the use of “Joint Inflammation model” instead of RA model, based on the published characterization and use of this model and have added 3 references to clarify the differences between the CFA model and RA models: *“To further delineate the molecular and cell biological pathways in intestinal responses to joint inflammation, we used a rat model in which local joint inflammation is induced by intra-articular injection (IAI) of Complete Freund’s adjuvant (CFA) [34, 35]. The predominant inflammatory response of a single injection of the adjuvant into a joint is a rapid development of synovial hyperplasia and joint swelling with immune cell infiltration (macrophages, neutrophils) [36]. The model is widely used to study pain behavior and intra-articular cytokine production and can produce changes in the intestinal microbiome [37, 38]. It should be noted that although joint inflammatory parameters are similar to RA, the model lacks RA characteristics, such as development of systemic T-cell driven autoimmunity or polyarthritis (PMID: 29695052; PMID: 20349327 PMID: 26539560). We used this model in the context of our objective to determine whether inflammatory changes in the CFA-exposed synovium induced temporally related changes in the intestinal barrier of the ileum and/or the colon.”*

We also alluded to the ‘self-limiting’ synovial inflammation in this model in the **Discussion pg. 14:** “Re-establishment of a smooth periosteal surface by 14d supports the conclusion that the specific macrophage-mediated inflammatory joint tissue responses in the CFA-model are self-limiting, which is further supported by a robust increase in transcripts for oxidative phosphorylation[126].”

**Querry 2.** In the phrase “However, it remains to be determined if the intestinal responses are mediated by a disrupted systemic immune response in RA, and/or if the bone and cartilage destruction mediated by an inflamed synovium (“RA pannus”) is the central link driving the intestinal pathogenesis in RA.” the authors are trying to suggest that systemic or localized immune response contribute to RA pathogenesis?

**Response:** We have modified the statement for clarification on pg 3:

*“However, it remains to be determined if the intestinal responses in the RA animal model are mediated by RA- specific systemic autoimmune responses, and/or if they develop in response to bone and joint tissue destruction mediated by an inflamed synovium (“RA pannus”).*

**Query 3.** The fluid of joints was recovered using PBS in a rinse. Can this procedure dilute the collected fluid? Besides, the manuscript does not clearly specify the volume of synovial fluid collected, which is crucial for interpreting protein concentration data. ---

**Response:** please see Reviewer 1 Query 6:

a) We have modified the description in the Methods for synovial fluid collection.

*“To retrieve synovial fluid the joint cavity was rinsed twice with 150µL 1xPBS and collected into sterile microcentrifuge tubes. Lavages from right and left knee joints from each individual rat were combined and centrifuged at 3,000 rpm/805 g for 10 min at 4°C to remove cells and debris. Supernatants were stored at -80ºC until further analyses.”*

b) Since all lavages were 150 uL volume, there is no differential dilution of proteins between animals. Moreover, we have interpreted our data in Figure 1 (increase in bikunin in Synovial Fluid) in correlation with data in Supplemental Figure S2 (elevated protein content in synovial fluid at days). We have modified the text in the Results Section Pg. 5 to clarify

*“A transient increase in serum protein effusion into the joint space was confirmed by increased in synovial fluid contents of serum-derived bikunin•CS as well as in both inter-α-inhibitor (IαI) and pre-α-inhibitor (PαI)* ***(Fig. 1B & Fig. S2 B, C)*** *Furthermore, an concurrent abundance of albumin and IgG in synovial fluids at 3d post-CFA-IAI (****Fig. S2D****), as determined by SDS-PAGE confirmed the accumulation of serum derived components in the joint space. However, since these proteins were no longer present in the synovial fluids collected at 14d post-CFA, but increased knee diameters persisted, the latter was likely due to joint tissue responses, such as hyperplasia and fibrotic remodeling of the synovium and joint capsule, as well as patellar tendon swelling (****Figs. 1D-I****). No alteration in joint swelling, joint fluid composition or tissue remodeling were noted after PBS-IAI. Notably, a concurrent decrease in all these species in the serum (****Fig. 1C****) at the peak inflammation times suggested that the liver did not respond by upregulating production of these acute phase proteins in this model.”*

**Query 4:** What is the abbreviation FFPE?

**Response:** We have included the definition for FFPE in Results section, pg 5**:** *“Coronal formalin fixed paraffin embedded (FFPE) sections*

**Query 5:** Please, indicate where is possible, the “n” of animals used in each experiment, mainly in the figure legends.

**Response:** The total n of animals used is listed in the Supporting Figure 1B for total n per group per experiment. We added a table to Supporting Figure 1, as C that now lists each outcome measure and the n per experiment. Copied below for ease of review:

**Query 6:** CD68 is a general marker of macrophages. Did the authors could illustrate these cells with a more specific marker to define them as M1 or M2 macrophage. The same can be imagined to T cells (Th1 or Th2).

**Response:** We have added a sentence to the Discussion Page to address such characterization in future work on this topic: ***“****Moreover, additional detailed characterization of the myeloid cells (M1, M2 macophages) accumulated in synovium and intestinal lining will be included in future experimentation to delineate mechanism of synovial pathotype in eliciting an intestinal response.”*

NOTE: Whilst we value the reviewers comment re the more detailed characterization of the myeloid cells accumulated in target tissues such as synovium and intestinal lining, this topic is outside the scope of the current work and would require additional costly experiments. Such identification will be included in future experimentation to delineate mechanism of synovial inflammation type in eliciting an intestinal response. We are fully aware of the availability of commercial antibodies commonly used to identify M1 and M2 macrophages. However, it is important to note that M1 and M2 are functional phenotypes, not distinct cell types and hence there is no single marker antibody that is used to identify one or the other macrophage subtype rather, a panel of antibodies in combination with flow cytometry is typically used to quantify abundance of M1 and M2 macrophages.

**Query 6:** I think the correct in the first paragraph of page 12 is Fig. 11C.

**Response**: Thank you for pointing this out; it has been corrected in the text.

**Query 7:** In the phrase “Our data contrast with the widespread intestinal manifestations observed in the chronic rodent models of inflammatory arthritis induced by immunization with type 2 collagen” the authors discuss the difference of results between this study and literature, emphasizing the models of chronic inflammations used by other studies. Could not the authors use references about experimental RA?

**Response:** We have added 2 pertinent references on pg 15: “Our data contrast with the widespread intestinal manifestations observed in the chronic rodent models of inflammatory arthritis induced by immunization with type 2 collagen[131 PMID: 32332732, PMID: 38503474].”

**Query 8:** The study is described as a mono-joint model, yet the systemic implications (e.g., gut responses) are emphasized. This raises questions about whether the observed intestinal changes are truly systemic or locally mediated via neural or humoral pathways.

**Response:** **We value the reviewer’s comment. We have indeed pointed to neural pathway involvement in the neural (pain) transmission of joint inflammation to elicit an intestinal response on pg. 16 in the Discussion:** “In this study, it likely was indicated in the development of gut inflammation and dysbiosis in response to psychological stressors [144], such as pain-induced anxiety [145], and could also underlie the presence of the allostatic tissue response [146]. …….suggesting that in future mechanistic studies for joint-gut communications, potential Vagus/ENS responses should be assessed”.

**We have also added the following statement:**

“Although systemic inflammatory auto-antibodies or T and B cell responses have not been detected in IA CFA rodent models, locally mediated humoral reactions might also contribute to the inter-organ responses seen here.”

**Query 9:** While the authors claim minimal changes in microbiome composition, the analysis appears superficial. More detailed taxonomic and functional profiling would strengthen this claim.

**Response:** We thank the reviewer for this comment. However, a more detailed analysis of the gut microbiome using “shotgun sequencing” approaches is outside the scope of this study but will be considered for future experiments.

**Query 10:** The study relies heavily on transcriptomic data. Functional assays (e.g., permeability tests, cytokine quantification) would validate the proposed allostatic mechanisms.

**Response:** We thank the reviewer for raising this point. However it is outside the scope of the reported study. Approaches to include in vivo functional assays for analyses of gut tissue responses are being developed for our ongoing studies of this topic. We have added a sentence in the Discussion pg 15: “Effects of the above responses on intestinal functionality in the CFA model will need additional evaluations including intestinal permeability assessment (21) and metabolomics studies (PMID: 34537381).”

**Query 11:** Finally, a better clarification in the discussion section about the response of the gut to systemic inflammation caused by RA or to localized joint signals will enrich the manuscript.

**Response:** Please see our modifications under Queries 7-10.
